# Supplementary material for: Asymmetric Synthesis of Quaternary Hydantoins via a Palladium-Catalyzed Aza-Heck Cyclization
Source: J Am Chem Soc. 2025 Nov 14;147(49):44692–8. doi: 10.1021/jacs.5c16022 (PMC12703750; doi:10.1021/jacs.5c16022)
Supplement: Supplementary file 2 [file ja5c16022_si_002.zip › All NMR FID Files/7/7_ProtonNMR/1/pdata/1/email_EM05-258-1-F30-44-1H_1_1.pdf]

qPROTON CDCl3 /opt/nmrdata emeck 2

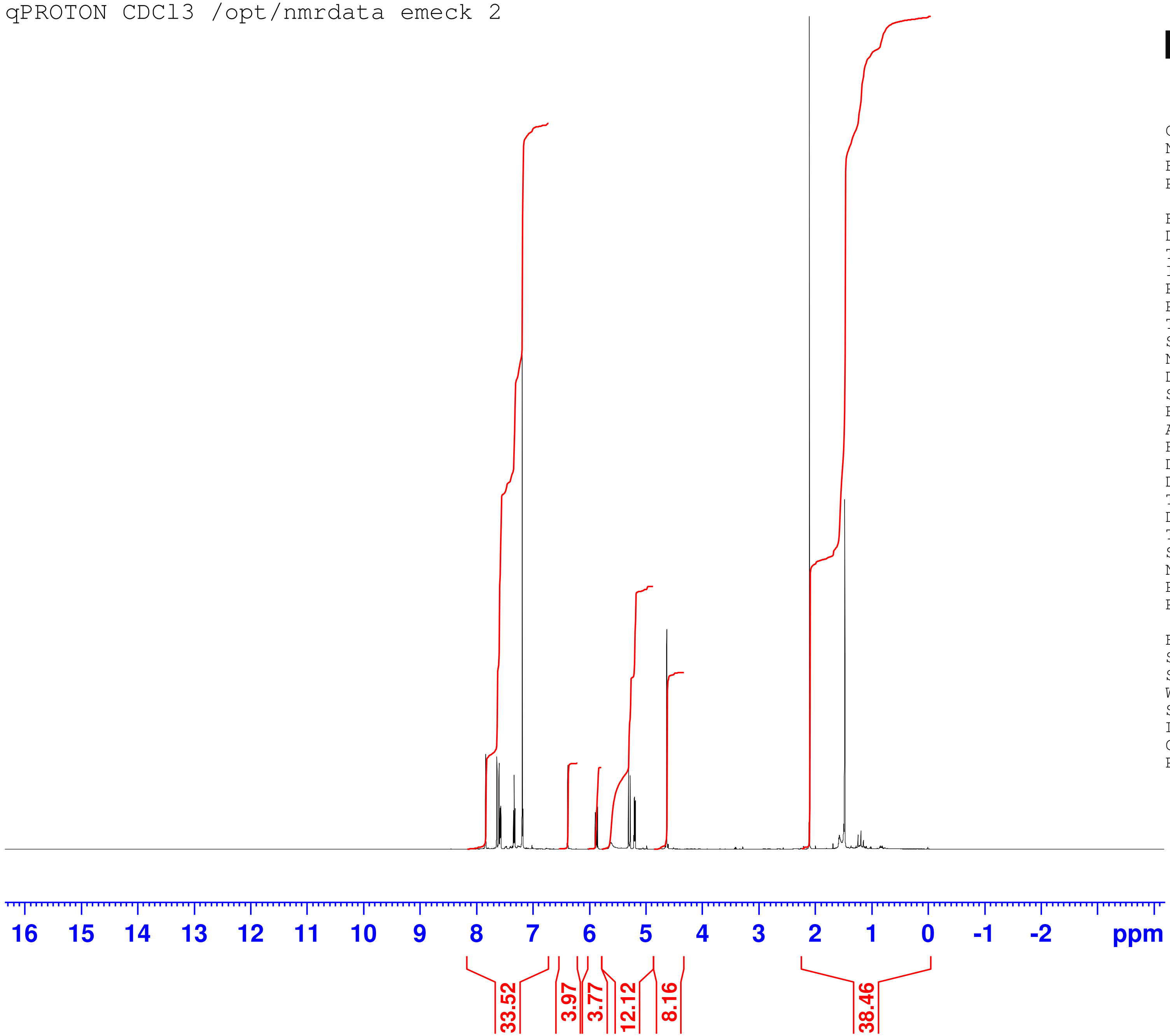


Current Data Parameters

| | |
|---|---|
| NAME | EM05-258-1-F30-44-1H |
| EXPNO | 1 |
| PROCNO | 1 |

F2 - Acquisition Parameters

| | |
|---|---|
| Date_ | 20241007 |
| Time | 9.25 h |
| INSTRUM | spect |
| PROBHD | Z168773_0053 ( |
| PULPROG | zg |
| TD | 65536 |
| SOLVENT | CDCl3 |
| NS | 16 |
| DS | 2 |
| SWH | 12335.526 Hz |
| FIDRES | 0.376450 Hz |
| AQ | 2.6563926 sec |
| RG | 64 |
| DW | 40.533 usec |
| DE | 18.00 usec |
| TE | 298.0 K |
| D1 | 10.00000000 sec |
| TD0 | 1 |
| SFO1 | 600.3237072 MHz |
| NUC1 | 1H |
| P1 | 12.00 usec |
| PLW1 | 21.85499954 W |

F2 - Processing parameters

| | |
|---|---|
| SI | 65536 |
| SF | 600.3200552 MHz |
| WDW | EM |
| SSB | 0 |
| LB | 0.30 Hz |
| GB | 0 |
| PC | 1.00 |
